# Values of multiparametric and biparametric MRI in diagnosing clinically significant prostate cancer: a multivariate analysis

**DOI:** 10.1186/s12894-024-01411-0

**Published:** 2024-02-16

**Authors:** Xiao Feng, Xin Chen, Peng Peng, He Zhou, Yi Hong, Chunxia Zhu, Libing Lu, Siyu Xie, Sijun Zhang, Liling Long

**Affiliations:** 1 Department of Radiology, The First Affiliated Hospital of Guangxi Medical University, No.6 Shuangyong Road, Qingxiu District, Nanning, 530021 Guangxi P.R. China; 2 Department of Radiology, Jiangjin Hospital, Chongqing University, No.725, Jiangzhou Avenue, Dingshan Street, Chongqing, 402260 China

**Keywords:** MpMRI, BpMRI, PI-RADSv2.1, csPCa, Multivariate analysis

## Abstract

**Background:**

To investigate the value of semi-quantitative and quantitative parameters (PI-RADS score, T2WI score, ADC, Ktrans, and Kep) based on multiparametric MRI (mpMRI) or biparametric MRI (bpMRI) combined with prostate specific antigen density (PSAD) in detecting clinically significant prostate cancer (csPCa).

**Methods:**

A total of 561 patients (276 with csPCa; 285 with non-csPCa) with biopsy-confirmed prostate diseases who underwent preoperative mpMRI were included. Prostate volume was measured for calculation of PSAD. Prostate index lesions were scored on a five-point scale on T2WI images (T2WI score) and mpMRI images (PI-RADS score) according to the PI-RADS v2.1 scoring standard. DWI and DCE-MRI images were processed to measure the quantitative parameters of the index lesion, including ADC, Kep, and Ktrans values. The predictors of csPCa were screened by logistics regression analysis. Predictive models of bpMRI and mpMRI were established. ROC curves were used to evaluate the efficacy of parameters and the model in diagnosing csPCa.

**Results:**

The independent diagnostic accuracy of PSA density, PI-RADS score, T2WI score, ADCrec, Ktrans, and Kep for csPCa were 80.2%, 89.5%, 88.3%, 84.6%, 58.5% and 61.6%, respectively. The diagnostic accuracy of bpMRI T2WI score and ADC value combined with PSAD was higher than that of PI-RADS score. The combination of mpMRI PI‑RADS score, ADC value with PSAD had the highest diagnostic accuracy.

**Conclusions:**

PI-RADS score according to the PI-RADS v2.1 scoring standard was the most accurate independent diagnostic index. The predictive value of bpMRI model for csPCa was slightly lower than that of mpMRI model, but higher than that of PI-RADS score.

## Background

Prostate cancer (PCa) is the second most common male cancer with the highest incidence in Western countries [1]. Multiparametric magnetic resonance imaging (mpMRI) is an efficient non-invasive tool for the diagnosis, staging, and monitoring of PCa [2]. The prostate imaging reporting and data system (PI-RADS) is a 5-point scale used to predict the possibility of clinically significant prostate cancer (csPCa) based on the findings of mpMRI, which includes T2-weighted imaging (T2WI), diffusion weighted imaging (DWI), and dynamic contrast-enhanced (DCE) [3]. However, its diagnosis is based on the subjective and semi-quantitative results of mpMRI. The latest PI-RADS v2.1which update of PI-RADS v2.0 in 2019, still does not incorporate clinical data and quantitative parameters, and shows no significant value in DEC imaging. Moreover, the current system does not cover suggestions for PI-RADS category 3 lesions and MRI follow-up [3, 4]. Studies have found no significant difference in the diagnostic efficiency for csPCa between biparametric magnetic resonance imaging (bpMRI) and mpMRI [5, 6]. Use of the combination of PI-RADS scores, patient’s age, prostate specific antigen (PSA) level, and prostate specific antigen density (PSAD) has been shown to increase the detection rate of csPCa, thus providing a more evaluable reference for clinical decision-making [7, 8]. In the present study, the subjects were assigned into csPCa group and non-csPCa group based on the pathological findings. Regarding the limitations of PI-RADSv2.1, we assessed the csPCa-predicting potential of the biparametric and multiparametric models, involving the semi-quantitative and quantitative parameters of mpMRI and bpMRI (e.g., PI-RADS scores and T2 weighted image [T2WI] score according to the latest PI-RADS v2.1 scoring standard, apparent diffusion coefficient [ADC], volume transfer constant between blood plasma and the extracellular extravascular space [K^trans^], rate constant between the extracellular extravascular space and the blood plasma [K_ep_]) and clinical parameters (PSAD).

## Material and methods

### Patients

**Fig. 1:**
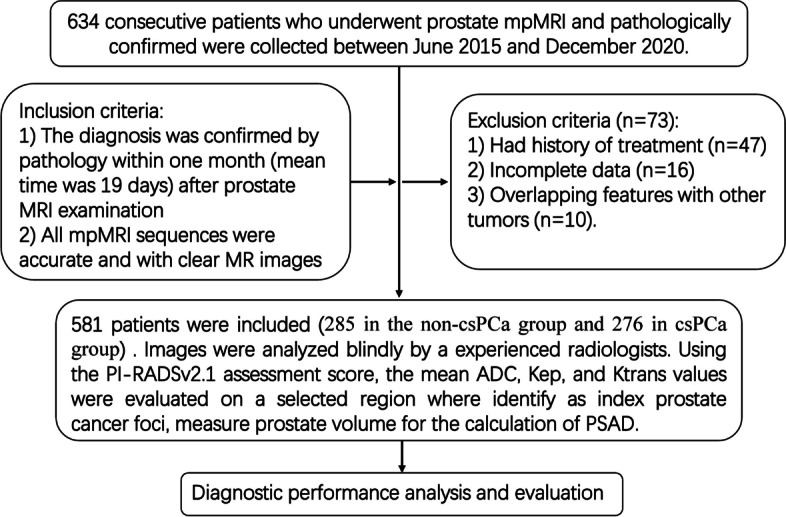
Study flowchart shows patient inclusions and exclusions

Since 2019, the imaging and clinical data were retrospectively collected from 634 patients who underwent prostate mpMRI at our hospital due to the increase in PSA level, and were confirmed by prostate biopsy or RP(198, 31%) between June 2015 and December 2020. The mpMRI was performed before or four weeks after biopsy to minimize the effect of artefacts induced by blood pooling within the gland. A total of 561 patients (age range 28–92 years; median 67 years) were included after excluding those who had history of treatment, incomplete data, such as PSA without specific value(> 100 ng/ml), or overlapping features with other tumors. Of them, 285 (50.8%) were assigned to the non-csPCa group and 276 (49.2%) to the csPCa group. The study flowchart is presented in Fig. 1.

This study was a retrospective cohort study approved by the institutional review board and complied with HIPAA. The requirement for informed consent was waived off by the human investigation committee at our institution.

### MRI Protocol

MRI was performed with a 3.0-T scanner (Siemens MAGNETOM Verio or Prisma, German) using a 16-channel phased-array body coil. The protocol included axial and sagittal T2WI, axial DWI and DCE in accordance with PI-RADS v2.1. b-values used for DWI included 0, 1000, and 2000 mm/s^2^. ADC map was automatically calculated from b-values of 0 and 1000 mm/s^2^. The MRI scanning parameters are presented in Tables 1 and 2.

**Table 1:** Multi-parametric MRI performed using Verio

	T1WI	T2WI/FS-T2WI	DWI	DCE
TR, ms	700	2090	9000	5.97
TE, ms	11	76	86	2.12
Slice-thickness/gap, mm	4/0.4	4/0.4	4/0.4	3/0.6
NEX	1	1	4	1
Matrix	256 × 256	256 × 256	111 × 172	135 × 192
FOV, mm^2^	180 × 180	180 × 180	224 × 260	200 × 180
voxel, mm^3^	0.7 × 0.7 × 0.4	0.7 × 0.7 × 0.4	2.0 × 1.5 × 4.0	1.5 × 1.0 × 3.0
Acquisition time, min	1:29	1:29	2:53	4:28

Verio stands for Siemens MAGNETIC resonance scanner model, *TR* Repetition time, *TE* Echo time, *NEX* Number of excitation, FOV Field of view

**Table 2:** Multi-parametric MRI performed using prisma

	T2WI/FS-T2WI	DWI	Zoomit-DWI	DCE
TR, ms	6980	4000	4300	6.24
TE, ms	104	63	69	2.1
Slice-thickness /gap, mm	3.5/0	3/0	3/0	2/0
NEX	2	4	2,3,9,9	1
Matrix	384 × 384	110 × 110	90 × 90	154 × 192
FOV, mm^2^	200 × 200	220 × 220	73 × 150	220 × 260
voxel, mm^3^	0.5 × 0.5 × 3.0	1.0 × 1.0 × 3.0	0.8 × 0.8 × 3.0	1.4 × 1.4 × 2.0
Acquisition time, min	3:22/2:28	4:06	5:14	5:01

Prisma stands for Siemens MAGNETIC resonance scanner model, *TR* Repetition time, *TE* Echo time, *NEX* Number of excitation, *FOV* Field of view

### Image analysis

Prostate volume was measured according to the PI-RADS v2.1 [3] standard for the calculation of PSAD. Prostate index lesions of each patient were scored on a five-point scale on T2WI images (T2WI score) and mpMRI images (PI-RADS score) by a senior radiologist blinded to the pathological results and having 12 years of MRI experience (having read more than eighty thousand patients’ MRI images, these included about 1,500 prostate MRI), according to the PI-RADS v2.1 scoring standard.

**Fig. 2:**
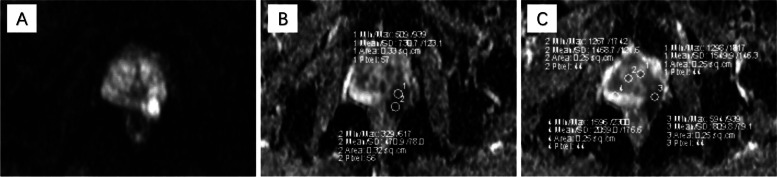
ADC measurements of prostate cancer. **A** shows an abnormally high signal focus in the left peripheral zone on the DWI (B-value 1000 mm/s^2^); **B**, **C** show that the mean ADC value of the lesion in the left peripheral zone is 0.670 × 10^-3^mm^2^/s

**Fig. 3:**
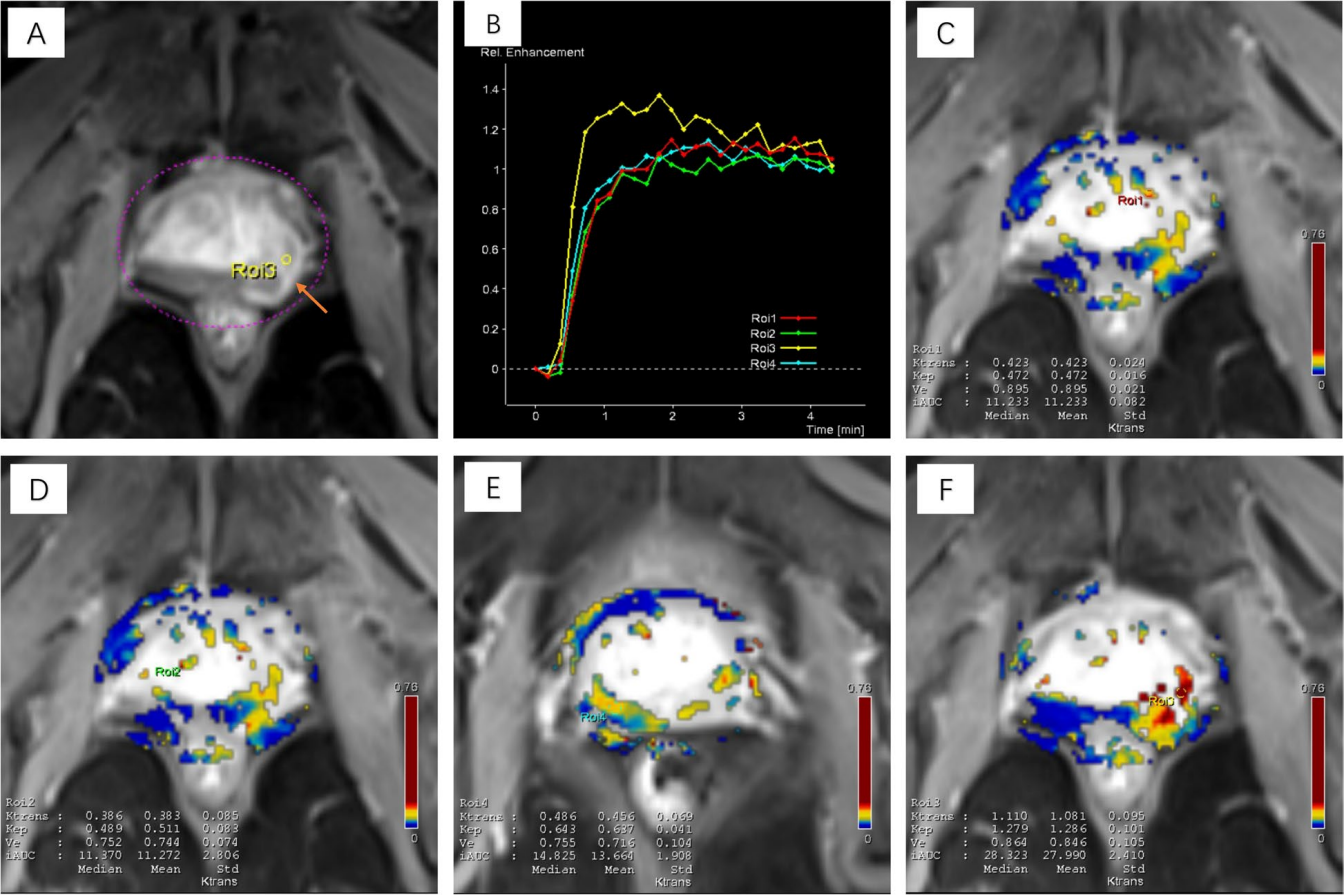
Parameter diagram of DCE measurements in prostate cancer. **A** Early dynamic contrast enhanced image shows avid enhancement within the anterior lesion (arrows). **B** The signal intensity-time curve of ROI3 shows a plateau of rapid rise and slow fall, while ROI1.2.4 shows an inflow curve of slow rise. **E** The Ktrans and Kep values of the index lesion are 1.110/min and 1.279/min. **C**, **D**, **F** shows the Ktrans and Kep values in the normal area of bilateral transition zone and right peripheral zone

All images were sent to a workstation. Specific software was used to process DW and DCE images (4D-Tissu). A senior radiologist analyzed all the mpMRI images that qualified the inclusion criteria to identify index prostate cancer foci. The mean ADC, Kep, and Ktrans values were evaluated on a selected region of interest encompassing as much of the inner part of the lesion as possible without contacting the edges (Figs. 2 and 3).

### Reference standard

Each patient underwent both systematic biopsy (with an average of 12 random samples from the entire prostate gland) and target biopsy (with at least three samples obtained from each lesion identified by MRI). Target sampling was performed with an MRI/TRUS fusion, alternately using the cognitive technique or dedicated software, coupled with various commercially-available ultrasound tools. For patients undergoing radical prostatectomy (RP) after puncture, pathological results obtained after RP were used as the gold standard for diagnosis. Post-RP specimens were sectioned at 4–6 mm intervals, and stained with hematoxylin and eosin (H&E). A pathologist recorded the presence or absence of PCa, tumor location, and determined the tumor Gleason score (GS) of biopsy specimens. GS was calculated according to the 2014 International Society of Urological Pathology Modified Gleason Grading System [9]. The definition of csPCa was a tumor with GS ≥ 7, or GS = 3 + 3 plus tumor size ≥ 0.5 mL [10]. Tumor size was calculated from mpMR images, most commonly T2WI. When multiple foci of PCa were found, the focus with the highest GS was considered as the index lesion.

### Statistical analysis

According to the pathological results after biopsy, patients were divided into two groups, i.e., csPCa and non-csPCa. Between-group differences with respect to each parameter (PSA density, PI-RADS score, T2WI score, ADC, Ktrans, and Kep) were assessed using the Independent-samples U test. The predictors of csPCa were screened by logistics regression analysis. In case of multicollinearity, logistic regression analysis was performed using the likelihood ratio forward method to screen variables in the model. mpMRI and bpMRI predictive models were established and receiver operating characteristic (ROC) curves were plotted to evaluate the efficiency of each parameter and the model in diagnosing csPCa. The diagnostic performance was compared using the DeLong test. Two-tailed *P* values < 0.05 were considered indicative of statistical significance.

## Results

**Fig. 4:**
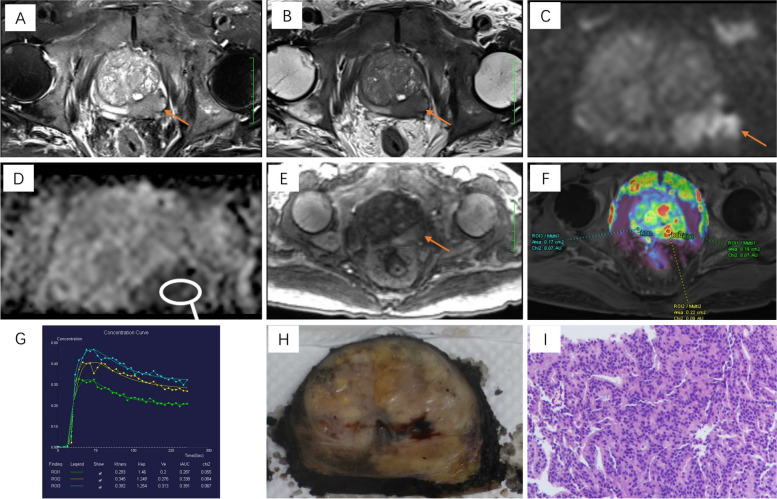
Representative case of prostate cancer An 80-year-old man with PSA 73.2 ng/mL, PSAD 0.77 ng/mL^2^, and Gleason score 3 + 4 prostate cancer confirmed after RP. **A**, **B** Axial T2WI and FS-T2WI sequences show a T2 hypointense nodule (arrows) involving the left peripheral zone with extraprostatic extension; T2WI score = 5. **C** Diffusion-weighted image (b = 2000) shows a markedly hyperintense signal (arrows) corresponding to (A) and (B). **D** ADC map image shows focal hypointense signal corresponding to (C), ADC value of the lesion is 0.829 × 10^-3^mm^2^/s; DWI PI-RADS = 5. **E** Early dynamic contrast enhanced image shows avid enhancement within the anterior lesion (arrows), DCE MRI PI-RADS = positive. PI-RADS score = 5. **F**, **G** The Ktrans and Kep values of the anterior lesion are 0.345/min and 1.249/min, respectively. **H** Gross morphology of the RP specimen. (I) Microscopic pathological view of the index lesion

All the 561 cases were confirmed by biopsies and eligible for this study. The non-csPCa group comprised of 285 patients, including 168 with benign prostatic hyperplasia (BPH), 49 with prostatitis, 20 with prostate intraepithelial neoplasia (PIN), and 48 with clinically insignificant Pca (ciPCa). The CsPCa group comprised of 276 patients, including 69 with International Society of Urological Pathology (ISUP) grade 2, 77 with ISUP grade 3, 60 with ISUP grade 4, and 70 with ISUP grade 5 prostate cancer (Fig. 4).

### Normality test of semi-quantitative and quantitative parameters

The one-sample Kolmogorov–Smirnov test showed non-normal distribution of PI-RADS score, T2WI score, ADC, K^trans^, K_ep_, PSAD, and patient’s age (*P* < 0.001 for all).

### Mann–Whitney U test of semi-quantitative and quantitative parameters

The csPCa group had significantly higher PSAD, PI-RADS score, T2WI score, K^trans^ and K_ep_, but significantly lower ADC compared to the non-csPCa group (*P* < 0.05 for all). No significant difference in age was detected between csPCa and non-csPCa group (*P* = 0.099, Table 3).

**Table 3:** Semi-quantitative and quantitative parameters in csPCa and non-csPCa group

Parameter	Non-csPCa	csPCa	U	*P*
PSAD (ng/mL^2^)	0.124 (0.069, 0.250)	0.280 (0.076, 739)	15,350.000	< 0.001
Age (years)	67 (62, 72)	68 (62, 74)	36,161.500	0.099
PI-RADS (points)	2 (1, 3)	5 (4, 5)	8256.500	< 0.001
T2WI (points)	2 (2, 3)	5 (4, 5)	9204.500	< 0.001
ADC (× 10^−3^mm^2^/s)	1.024 (0.851, 1.200)	0.680 (0.577, 0.779)	12,627.000	< 0.001
K_ep_ (/min)	1.390 (1.014, 1.980)	1.893 (1.125,2.619)	30,359.500	< 0.001
K^trans^ (/min)	1.018(0.660, 1.414)	1.264(0.674, 1.830)	32,728.500	< 0.001

*csPCa* clinically significant prostate cancer, *PSAD* Prostate specific antigen density, *PI-RADS* Prostate imaging reporting and data system, *T2WI* T2 weighted image, *ADC* Apparent diffusion coefficient, *Ktrans* volume transfer constant between blood plasma and the extracellular extravascular space, *Kep* rate constant between the extracellular extravascular space and the blood plasma. Data are expressed as M (Q1, Q3)

### Univariable logistic regression analysis of semi-quantitative and quantitative parameters and their respective diagnostic efficiency

**Fig. 5:**
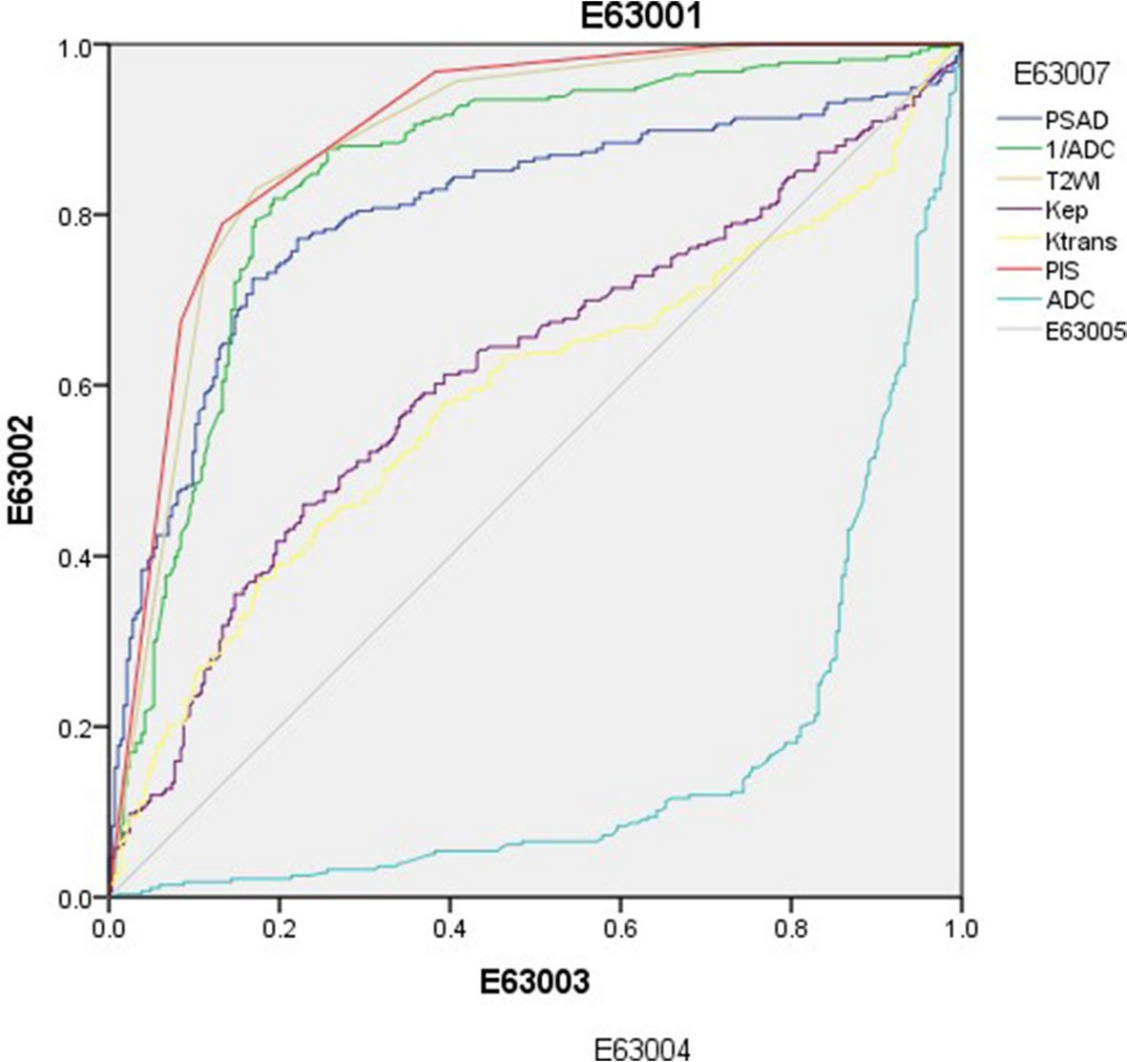
ROC curve of each variable for independent diagnosis of csPCa

Univariable logistic regression analysis showed significant differences between csPCa and non-csPCa groups with respect to PI-RADS score, T2WI score, ADC, and PSAD (*P* < 0.05 for all), but not with respect to K^trans^, K_ep_ and ADC reciprocal (ADCrec). Furthermore, receiver operating characteristic (ROC) curve analysis revealed that PI-RADS score showed the highest diagnostic efficiency for csPCa, followed by T2WI score, ADCrec, PSAD, K_ep,_ and K^trans^, in that order (*P* = 0.000 for all, Fig. 5, Table 4).

**Table 4:** Univariable logistic regression and ROC curve analysis of semi-quantitative and quantitative parameters in diagnosing csPCa

	OR	95% CI for OR		*P* value	AUC	95% CI for AUC	
		Lower	Upper			Lower	Upper
PIS	2.163	1.610	2.906	.000	.895	.868	.922
T2WI	1.745	1.315	2.314	.000	.883	.854	.912
PSAD	1.535	1.153	2.042	.003	.802	.764	.841
ADC	.111	.016	.784	.028	.154	.120	.188
K_ep_	.987	.698	1.395	.940	.616	.569	.663
K^trans^	.794	.449	1.403	.428	.585	.537	.633
ADCrec	.812	.326	2.023	.654	.846	.812	.880

*PIS* The 5-point results of PI-RADS v2.1 assessed by senior physicians, *T2WI* The 5-point results of T2WI, *PSAD* Prostate specific antigen density, *ADCrec* Reciprocal of apparent diffusion coefficient, *OR* Odds ratio (OR > 1 indicated that the parameter was a risk factor; otherwise, it was a protective factor), *CI* Confidence interval, *AUC* Area under the curve

Kendall’s tau-b correlation coefficient showed a significant correlation between PI-RADS score and T2WI score (*t* = 0.769, *P* < 0.001). To avoid multicollinearity among variables, PI-RADS score and T2WI score were separately introduced into the biparametric and multiparametric models.

### Multivariable logistic regression analysis of Semi-quantitative and quantitative parameters

The biparametric model involving ADC measured by bpMRI plus T2WI scores and PSAD, and the multiparametric model involving ADC measured by mpMRI plus PI-RADS score and PSAD were created by binary logistic regression.

The first biparametric model involving ADC measured by bpMRI plus T2WI score and PSAD is shown below:

$Logit \left(P\right)= -1.925+0.494\times PSAD+1.006 \times T2WI\,scores-2.434 \times ADC\ ;$ in which, the independent variables, including T2WI score (OR = 2.734; 95% CI, 2.199–3.398), ADC (OR = 0.088; 95% CI, 0.029–0.266), and PSAD (OR = 1.639, 95% CI, 1.223–2.197) were all statistically significant (*P* < 0.05 for all).

The model could predict 83.8% of csPCa cases, with a positive predictive value of 83.4% and a negative predictive value of 84.2% (χ^2^ = 363.055, *P* < 0.001).

The second multiparametric model involving ADC measured by mpMRI plus PI-RADS score and PSAD is shown below:

$Logit \left(P\right)= -2.212+0.441\times PSAD+1.120 \times PI-RADS\, scores-2.350 \times ADC\ ;$ in which, the independent variables, including PI-RADS scores (OR = 3.064; 95% CI, 2.428–3.866), ADC (OR = 0.095; 95% CI, 0.032–0.288), and PSAD (OR = 1.554, 95% CI, 1.170–2.064) were all statistically significant (*P* < 0.05 for all).

The model predicted 85.2% of csPCa cases with a positive predictive value of 86.4% and a negative predictive value of 84.1% (χ^2^ = 376.368, *P* < 0.001).

### Diagnostic efficiency of the biparametric and multiparametric models in diagnosing csPCa compared with that of PI-RADS

**Fig. 6:**
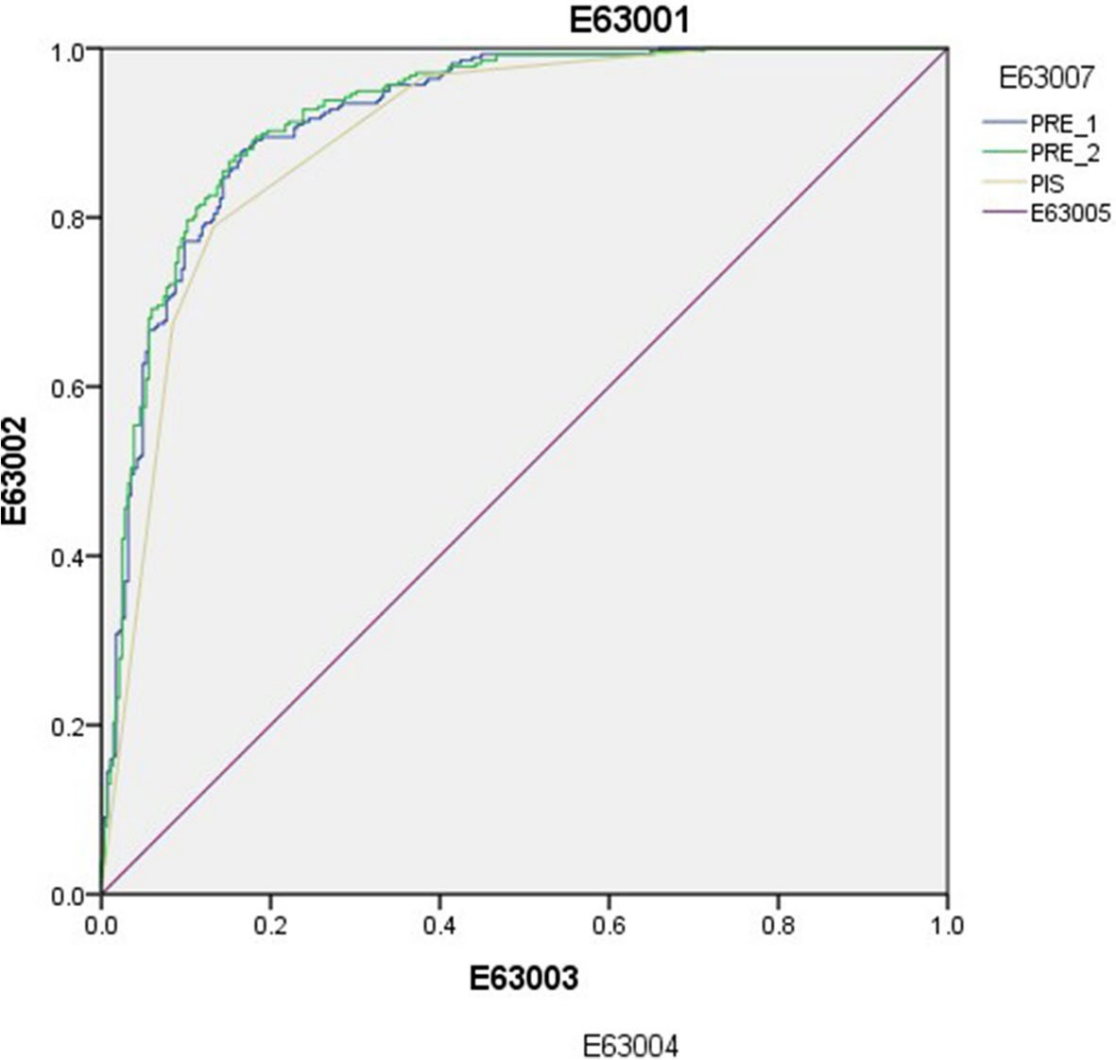
ROC curve of each model and PI-RADS score in the diagnosis of csPCa PIS, the 5-point results of PI-RADS v2.1 assessed by senior physicians; PRE1, the biparametric model involving ADC measured by bpMRI plus T2WI scores and PSAD; PRE2, the multiparametric model involving ADC measured by mpMRI plus PI-RADS scores and PSAD; ROC, receiver operating characteristic; PI-RADS, prostate imaging reporting and data system

The Areas under curve (AUC) of the multiparametric model was significantly higher than those of the biparametric model and PI-RADS (Delong test *P* < 0.05, Fig. 6). The multiparametric model showed the highest Youden index, followed by the biparametric model (Table 5).

**Table 5:** Diagnostic efficiency of PI-RADS, biparametric model, and multiparametric model for csPCa

	PIS	PRE1	PRE2
AUC (95% CI)	0.895 (0.868–0.922)	0.918 (0.893–0.940)	0.923 (0.898–0.944)
Cut-off value	> 3	> 0.436	> 0.390
Sensitivity (%)	78.99%	88.0%	87.32%
Specificity (%)	86.67%	83.2%	84.21%
Youden index	0.657	0.712	0.715
*P* value			
PI-RADS	-	< 0.0001	< 0.0001
PRE1	< 0.0001	-	0.0202
PRE2	< 0.0001	0.0202	-

*PI-RADS* Prostate imaging reporting and data system, *csPCa* clinically significant prostate cancer, *AUC* Area under the curve, *PIS* the 5-point results of PI-RADS v2.1 assessed by senior physicians, *PRE1* the biparametric model involving ADC measured by bpMRI plus T2WI scores and PSAD, *PRE2* the multiparametric model involving ADC measured by mpMRI plus PI-RADS scores and PSAD; “- “ indicates not applicable

Thirty cases were selected to verify the accuracy of the prediction model. The sigmoid function was used for the conversion. The average *P* values (the probability of csPCa occurrence) were 69.87% and 71.36% in the csPCa group, and 16.22% and 15.31% in the non-csPCa group. The top 10 representative ROI verification results are presented in Table 6.

**Table 6:** Logistic regression equation verification results

sample	PSAD	T2WI	ADC	PI-RADS	P_b_	P_m_	pathology
1	0.102	1	1.314	1	1.68%	1.58%	BPH
2	0.082	2	1.122	2	6.89%	7.09%	BPH with inflammation
3	0.056	3	0.967	2	22.57%	9.80%	inflammation
4	0.045	4	1.035	3	40.18%	22.02%	PIN
5	0.349	3	0.669	3	41.02%	43.28%	Gleason 3 + 3
6	0.886	3	0.627	3	50.11%	51.63%	Gleason 3 + 4
7	0.243	3	0.675	4	39.41%	68.76%	Gleason 4 + 3
8	1.454	3	0.759	4	49.10%	75.50%	Gleason 3 + 5
9	3.255	5	0.433	5	97.49%	97.82%	Gleason 4 + 5
10	2.033	5	0.478	5	95.01%	95.93%	Gleason 5 + 5

*PSAD* Prostate specific antigen density, *T2WI* T2 weighted image, *ADC* Apparent diffusion coefficient, *PI-RADS* Prostate imaging reporting and data system, *Pb* the probability of csPCa occurrence calculated by bpMRI equation, *Pm* the probability of csPCa occurrence calculated by mpMRI equation

## Discussion

In the present study, the PI-RADS v2.1 based on the mpMRI findings showed relatively high accuracy (89.5%), sensitivity (78.9%), and specificity (86.6%) for diagnosis of csPCa, which is consistent with those findings in previous studies [11, 12]. According to a meta-analysis [12], the diagnostic sensitivity and specificity of PI-RADS v2.1 for csPCa were 87% and 74%, respectively. However, a study by Westphalen et al. [13] suggested relatively low positive predictive value of PI-RADS v2.1 for prostate MRI (49%; 95% CI, 40–58%) due to the strong subjectivity in the process of scoring. The independent diagnostic efficiency of quantitative parameters, like ADC (84.6%) and PSAD (80.2%), are relatively low, but these parameters are more objective and measurable, due to the well-recognized standards and measurement repeatability. Pepe et al. [14] found that ADC evaluation could support clinicians in decision making of patients with PI-RADS score 3 at risk for csPCa, for increase the ROC from 0.71 to 0.81. Marco [15] found that PSAD can help detected mpMRI false negative csPCa. And several studies have shown that PI-RADS combined with ADC or PSAD significantly enhances the diagnostic accuracy and positive predictive value of csPCa, thus avoiding unnecessary biopsy [16–18]. DCE MRI is an established mpMRI sequence for assessing prostate cancer, which highlights hemodynamic changes in cancer lesions and measures quantitative parameters that reflect microvascular perfusion (e.g., K_ep_, K^trans^) [19]. As a single predictor, the odds ratios (OR) of K_ep_ (0.987, *P* > 0.05) and K^trans^ (0.794, *P* > 0.05) in the present study were closer to 1, suggesting that they presented no contribution to the predictive model of csPCa, which is consistent with previous findings [20, 21]. We created predictive models based on MR imaging data, quantitative parameters, and clinical indicators, which not only significantly enhance the diagnostic accuracy for csPCa, but also objectively identify the cancer lesion. The results were similar with those from Liying Han [22]. Its area under curve value of the combined model (0.911) was also higher than those of ADC, PSAD, and PI-RADS v2.0 (0.887, 0.861, and 0.859, respectively).

In recent years, a large number of scholars have proposed that prostate MRI without DCE (bpMRI) may replace PI-RADSv2.1 based on mpMRI as a non-invasive monitoring means for csPCa [23–25]. Comparing the results of a meta-analyses [24], the diagnostic sensitivity (87%, 95%CI: 78%-93%) and specificity (72%, 95%CI: 56%-84%) of mpMRI for csPCa were not significantly different from bpMRI (sensitivity: 84%, 95%CI: 80% to 88%, specificity 75%, 95%CI: 68% to 81%). However, in our study, the diagnostic efficiency and positive predictive value of multiparametric model were significantly higher than those of the biparametric model. That’s consistent with PI-RADS v2.1, when bpMRI is performed and DCE data are not obtained, transition zone (TZ) assessment remains unchanged, and the proportion of men with PI-RADS assessment category 3 may increase [3]. Similarly, Greer et al. [26] found that DCE-MRI was conducive to enhance the diagnostic efficiency for csPCa, and the abnormal findings on DCE-MRI significantly increased the detection rate of PI-RADS v2.0 in categories 2–5 (A total of 163 patients with 654 lesions were evaluated). Therefore, mpMRI can be recommended to avoid misdiagnosis of csPCa, particularly suitable for prostate cancer risk groups.

Although the diagnostic efficiency of multiparametric model for csPCa was superior to that of the biparametric model, the complex procedure may challenge junior physicians or physicians in low-level hospitals with fewer cases. In the present study, the negative predictive value of the biparametric model was comparable to that of the multiparametric model (84.2% vs. 84.1%). Moreover, considering the risks associated with intravenous injection of contrast agents, and low economic and time cost of dynamic contrast-enhanced MRI, we think biparametric model might be more appropriate for early screening of csPCa.

This was a retrospective cohort study with an expanded sample size. Biopsy was not performed prior to mpMRI. Most of the involved subjects were pathologically diagnosed as prostate diseases by MRI/ultrasound fusion-guided biopsy or pathological examination after radical prostatectomy. This helped improve the reliability of our findings. In addition, this is first study to compare the characteristics and clinical value of the biparametric and multiparametric models involving MRI imaging data, quantitative parameters, and clinical indicators with those of PI-RADS v2.1. We identified the critical role of DCE-MRI in diagnosing csPCa, which can make up for the limitations of PI-RADS v2.1. Collectively, the biparametric and multiparametric models were found to be useful tools for selecting the optimal MRI and for planning the therapeutic strategy. However, its accuracy still requires to be verified by a larger sample from multiple centers. The latest research [27, 28] showed that MRI scoring with the Prostate Imaging for Recurrence Reporting assessment based on mpMRI could provide structured, reproducible, and accurate evaluation of local recurrence after definitive therapy for prostate cancer. Meanwhile, Pepe’s study shows PSMA PET/CT demonstrated good accuracy in the diagnosis of csPCa, which was not inferior to mpMRI (77.5% vs. 73.7%) [29]. These will be the focus of further research.

Some limitations of our study should be acknowledged. First of all, this study focused on the diagnosis of csPCa without taking into consideration the stage of cancer lesions. We chose the index lesion of csPCa because it contains lethal progenitor cells that determine the progression and metastasis of prostate cancer [30, 31]. Second, there were no independent studies on the diagnostic efficacy of transitional zone and peripheral zone lesions in this study. Third, this was a single-center retrospective cohort study. Our results should be further validated in a larger, multi-center prospective study.

## Conclusions

PI-RADS score was the most accurate independent diagnostic index. The predictive value of bpMRI model for csPCa was slightly lower than that of mpMRI model, but higher than that of PI-RADS score. Created bpMRI and mpMRI models for diagnosing csPCa, can overcome the limitations of PI-RADS v2.1 and facilitate treatment decision-making. BpMRI might be more appropriate for early screening of csPCa, and mpMRI for avoiding missed diagnosis.

## Data Availability

The data and materials used during the current study are available from the corresponding author upon reasonable request.

## Abbreviations

PCa: Prostate cancer
mpMRI: Multiparametric magnetic resonance imaging
PI-RADS: Prostate imaging reporting and data system
csPCa: Clinically significant prostate cancer
DEC: Dynamic contrast-enhanced
bpMRI: Biparametric magnetic resonance imaging
PSAD: Prostate specific antigen density
TRUS: Transrectal Ultrasound
MRF-TB: MRI-US fusion targeted biopsy
RP: Radical prostatectomy
T2WI: T2 weighted image
ADC: Apparent diffusion coefficient
K^trans^: Volume transfer constant between blood plasma and the extracellular extravascular space
K_ep_: Rate constant between the extracellular extravascular space and the blood plasma
GS: Gleason score
ROC: Receiver operating characteristic
ciPCa: Clinically insignificant Pca
ISUP: International Society of Urological Pathology
AUC: Areas under curve
OR: Odds Ratios
